# Cost efficiency of institutional incentives for promoting cooperation in finite populations

**DOI:** 10.1098/rspa.2021.0568

**Published:** 2021-10

**Authors:** Manh Hong Duong, The Anh Han

**Affiliations:** ^1^ School of Mathematics, University of Birmingham, Birmingham B15 2TT, UK; ^2^ School of Computing, Engineering and Digital Technologies, Teesside University, Middlesbrough TS1 3BX, UK

**Keywords:** institutional incentives, evolutionary game theory, evolution of cooperation

## Abstract

Institutions can provide incentives to enhance cooperation in a population where this behaviour is infrequent. This process is costly, and it is thus important to optimize the overall spending. This problem can be mathematically formulated as a multi-objective optimization problem where one wishes to minimize the cost of providing incentives while ensuring a minimum level of cooperation, sustained over time. Prior works that consider this question usually omit the stochastic effects that drive population dynamics. In this paper, we provide a rigorous analysis of this optimization problem, in a finite population and stochastic setting, studying both pairwise and multi-player cooperation dilemmas. We prove the regularity of the cost functions for providing incentives over time, characterize their asymptotic limits (infinite population size, weak selection and large selection) and show exactly when reward or punishment is more cost efficient. We show that these cost functions exhibit a phase transition phenomenon when the intensity of selection varies. By determining the critical threshold of this phase transition, we provide exact calculations for the optimal cost of the incentive, for any given intensity of selection. Numerical simulations are also provided to demonstrate analytical observations. Overall, our analysis provides for the first time a selection-dependent calculation of the optimal cost of institutional incentives (for both reward and punishment) that guarantees a minimum level of cooperation over time. It is of crucial importance for real-world applications of institutional incentives since the intensity of selection is often found to be non-extreme and specific for a given population.

## 1. Introduction

The problem of promoting the evolution of cooperative behaviour within populations of self-regarding individuals has been intensively investigated across diverse fields of behavioural, social and computational sciences [1–5]. Various mechanisms responsible for promoting the emergence and stability of cooperative behaviours among such individuals have been proposed. They include kin and group selection [6,7], direct and indirect reciprocities [8–12], spatial networks [13–16], reward and punishment [17–22] and pre-commitments [23–27]. Institutional incentives, namely rewards for cooperation and punishment for wrongdoing, are among the most important ones [22,28–36]. Different from other mechanisms, in order to carry out institutional incentives, it is assumed that there exists an *external* decision maker (e.g. institutions such as the United Nations and the European Union) that has a budget to interfere in the population to achieve a desirable outcome. Institutional enforcement mechanisms are crucial for enabling large-scale cooperation. Most modern societies implement certain forms of institutions for governing and promoting collective behaviours, including cooperation, coordination and technology innovation [37–42].

Providing incentives is costly and it is therefore important to minimize the cost while ensuring a sustained level of cooperation over time [28,31,41]. Despite its paramount importance, so far there have been only a few works exploring this question. In particular, Wang *et al*. [35] use optimal control theory to provide an analytical solution for cost optimization of institutional incentives assuming deterministic evolution and infinite population sizes (modelled using replicator dynamics). This work therefore does not take into account various stochastic effects of evolutionary dynamics such as mutation and non-deterministic behavioural update [4,43,44]. In a deterministic system consisting of cooperators and defectors, once the latter disappear (for instance through strong institutional punishment), there is no further change to the system and thus no further interference in it is required. When mutation is present, this behaviour can however recur and become abundant over time, requiring institutions to spend more of their budget on providing further incentives. Moreover, a key factor of behavioural update, the intensity of selection [4]—which determines how strongly an individual bases their decision to copy another individual’s strategy on their fitness difference—might strongly impact an institutional incentives strategy and its cost efficiency. Its value is usually found to be specific for a given population [45–48] and thus should be taken into account when designing suitable cost-efficient incentives. For instance, when selection is weak such that behavioural update is close to a random process (i.e. an imitation decision is independent of how large the fitness difference is), providing incentives would make little difference to cause behavioural change, however strong it is. When selection is strong, incentives that ensure a minimum fitness advantage to cooperators would ensure a positive behavioural change.

In a stochastic, finite-population context, so far this problem has been investigated primarily using agent-based and numerical simulations [28,31,49–52]. Results demonstrate several interesting phenomena, such as the significant influence of the intensity of selection on incentive strategies and optimal costs. However, there is no satisfactory rigorous analysis available at present that allows one to determine the optimal way of providing incentives. This is a challenging problem because of the large but finite population size and the complexity of stochastic processes governing the population dynamics.

In this paper, we provide exactly such a rigorous analysis. We study cooperation dilemmas in both pairwise (the Donation game (DG)) and multi-player (the Public Goods game (PGG)) settings [4]. They are among the most well-studied models for investigating the evolution of cooperative behaviour where individual defection is always preferred over cooperation while mutual cooperation is the preferred collective outcome for the population as a whole. Adopting a popular stochastic evolutionary game approach for analysing well-mixed finite populations [53–55], we derive the total expected costs of providing institutional reward or punishment, characterize their asymptotic limits (namely, for an infinite population, weak selection and strong selection) and show the existence of a phase transition phenomenon in the optimization problem when the intensity of selection varies. We calculate the critical threshold of phase transitions and study the minimization problem when the selection is less than and greater than the critical value. We furthermore provide numerical simulations to demonstrate the analytical results.

The rest of the paper is organized as follows. In §2, we introduce the models and methods, deriving mathematical optimization problems that will be studied. The main results of the paper are presented in §3. In §4, we discuss possible extensions for future work. Finally, detailed computations, technical lemmas and proofs of the main results are provided in the electronic supplementary material.

## 2. Models and methods

### (a) Cooperation dilemmas

We consider a well-mixed, finite population of N self-regarding individuals or players, who interact with each other using one of the following one-shot (i.e. non-repeated) cooperation dilemmas: the DG or its multi-player version, the PGG. In these games, a player can choose either to cooperate (i.e. a cooperator or C player) or to defect (i.e. a defector, or D player).

Let $\Pi_{C}(i)$ and $\Pi_{D}(i)$ be the average pay-offs of a C player and a D player in a population with i C players and N−i D players, respectively (see also §2.3 for more details). We show below that the difference $\delta =\Pi_{C}(i)-\Pi_{D}(i)$ does not depend on i. For cooperation dilemmas, it is always the case that δ<0.

#### (i) Donation game

The pay-off matrix of the DG (for a row player) is given as follows:

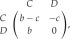

where c and b represent the cost and benefit of cooperation, where b>c. DG is a special version of the Prisoner’s Dilemma (PD) game.

Denoting $\pi_{X,Y}$ as the pay-off of a strategist X when playing with strategist Y from the pay-off matrix above, we obtain

$$\Pi_{C}(i)=\frac{(i-1)\pi_{C,C}+(N-i)\pi_{C,D}}{N-1}=\frac{\left(i-1)(b-c)+(N-i)(-c\right)}{N-1}$$

and

$$\Pi_{D}(i)=\frac{i\pi_{D,C}+(N-i-1)\pi_{D,D}}{N-1}=\frac{ib}{N-1}.$$

Thus,

$$\delta =\Pi_{C}(i)-\Pi_{D}(i)=-\left(c+\frac{b}{N-1}\right).$$

#### (ii) Public Goods game

In a PGG, players interact in a group of size n, where they decide to cooperate, contributing an amount c>0 to a common pool, or to defect, contributing nothing to the pool. The total contribution in a group will be multiplied by a factor r, where 1<r<n (for the PGG to be a social dilemma), which is then shared equally among all members of the group, regardless of their strategy.

We obtain [56]

ΠC(i)=∑j=0n−1(i−1j)(N−in−1−j)(N−1n−1)((j+1)rcn−c)=rcn(1+(i−1)n−1N−1)−c

and

ΠD(i)=∑j=0n−1(ij)(N−1−in−1−j)(N−1n−1)jrcn=rc(n−1)n(N−1)i.

Thus,

$$\delta =\Pi_{C}(i)-\Pi_{D}(i)=-c\left(1-\frac{r(N-n)}{n(N-1)}\right).$$

### (b) Cost of institutional reward and punishment

To reward a cooperator (respectively, punish a defector), the institution has to pay an amount θ/a (resp., θ/b) so that the cooperator’s (defector’s) pay-off increases (decreases) by θ, where a,b>0 are constants representing the efficiency ratios of providing the corresponding incentive. As we study reward and punishment separately, without losing generality, we set a=b=1 [22,28]. Thus, the key question here is: *What is the optimal value of the individual incentive cost* θ *that ensures a sufficient desired level of cooperation in the population (in the long run) while minimizing the total cost spent by the institution?*

#### (i) Deriving the expected cost of providing institutional incentives

We adopt here the finite population dynamics with the Fermi strategy update rule [44], stating that a player A with fitness $f_{A}$ adopts the strategy of another player B with fitness $f_{B}$ with a probability given by $P_{A,B}={\left(1+{\text{e}}^{-\beta (f_{B}-f_{A})}\right)}^{-1}$, where β represents the intensity of selection (see details in §2c). We compute the expected number of times the population contains i *C* players, 1≤i≤N−1. For that, we consider an absorbing Markov chain of (N+1) states, $\left\{S_{0},\ldots ,S_{N}\right\}$, where $S_{i}$ represents a population with i C players. $S_{0}$ and $S_{N}$ are absorbing states. Let $U={\left\{u_{ij}\right\}}_{i,j=1}^{N-1}$ denote the transition matrix between the N−1 transient states, $\left\{S_{1},\ldots ,S_{N-1}\right\}$. The transition probabilities can be defined as follows, for 1≤i≤N−1:

2.1 $$\begin{array}{rl} & u_{i,i\pm j}=0\;\text{for all }j\geq 2,\\ & u_{i,i\pm 1}=\frac{N-i}{N}\frac{i}{N}{\left(1+{\text{e}}^{\mp \beta [\Pi_{C}(i)-\Pi_{D}(i)+\theta ]}\right)}^{-1}\\ \text{and}\;\; & u_{i,i}=1-u_{i,i+1}-u_{i,i-1}. \end{array}\}$$

The entries $n_{ij}$ of the so-called fundamental matrix $N={\left(n_{ij}\right)}_{i,j=1}^{N-1}={\left(I-U\right)}^{-1}$ of the absorbing Markov chain give the expected number of times the population is in the state $S_{j}$ if it starts in the transient state $S_{i}$ [57]. As a mutant can randomly occur at either $S_{0}$ or $S_{N}$, the expected number of visits at state $S_{i}$ is, thus, $\frac{1}{2}(n_{1i}+n_{N-1,i})$.

The total cost per generation is

$$\theta_{i}=\left\{ \begin{array}{ll} i\times \theta & \text{in the case of institutional reward},\\ (N-i)\times \theta & \text{in the case of institutional punishment}. \end{array}\right.$$

Hence, the expected total costs of interference for institutional reward and institutional punishment are, respectively,

2.2 $$E_{r}(\theta )=\frac{\theta }{2}\sum_{i=1}^{N-1}(n_{1i}+n_{N-1,i})i\;\text{and}\;E_{p}(\theta )=\frac{\theta }{2}\sum_{i=1}^{N-1}(n_{1i}+n_{N-1,i})\;(N-i).$$

#### (ii) Cooperation frequency

Since the population consists of only two strategies, the fixation probabilities of a C (D) player in a homogeneous population of D (C) players when the interference scheme is carried out are, respectively,

$$\rho_{D,C}={\left(1+\sum_{i=1}^{N-1}\prod_{k=1}^{i}\frac{1+{\text{e}}^{\beta (\Pi_{C}(k)-\Pi_{D}(k)+\theta )}}{1+{\text{e}}^{-\beta (\Pi_{C}(k)-\Pi_{D}(k)+\theta )}}\right)}^{-1}$$

and

$$\rho_{C,D}={\left(1+\sum_{i=1}^{N-1}\prod_{k=1}^{i}\frac{1+{\text{e}}^{\beta (\Pi_{D}(k)-\Pi_{C}(k)-\theta )}}{1+{\text{e}}^{-\beta (\Pi_{D}(k)-\Pi_{C}(k)-\theta )}}\right)}^{-1}.$$

Computing the stationary distribution using these fixation probabilities, we obtain the frequency of cooperation (see §2.3),

$$\frac{\rho_{D,C}}{\rho_{D,C}+\rho_{C,D}}.$$

Hence, this frequency of cooperation can be maximized by maximizing

2.3 $$\max_{\theta }(\rho_{D,C}/\rho_{C,D}).$$

The fraction in equation (2.3) can be simplified as follows [54]:

2.4 $$\begin{array}{rl} \frac{\rho_{D,C}}{\rho_{C,D}} & =\prod_{k=1}^{N-1}\frac{T^{-}(k)}{T^{+}(k)}=\prod_{k=1}^{N-1}\frac{1+{\text{e}}^{\beta [\Pi_{C}(k)-\Pi_{D}(k)+\theta ]}}{1+{\text{e}}^{-\beta [\Pi_{C}(k)-\Pi_{D}(k)+\theta ]}}\\ & ={\text{e}}^{\beta \sum_{k=1}^{N-1}(\Pi_{C}(k)-\Pi_{D}(k)+\theta )}\\ & ={\text{e}}^{\beta (N-1)(\delta +\theta )}. \end{array}$$

In the above transformation, $T^{-}(k)$ and $T^{+}(k)$ are the probabilities of decreasing or increasing the number of *C* players (i.e. k) by one in each time step, respectively.

We consider non-neutral selection, i.e. β>0 (under neutral selection, there is no need to use incentives). Assuming that we desire to obtain at least an ω∈[0,1] fraction of cooperation, i.e. $\rho_{D,C}/\left(\rho_{D,C}+\rho_{C,D}\right)\geq \omega$, it therefore follows from equation (2.4) that

2.5 $$\theta \geq \theta_{0}(\omega )=\frac{1}{(N-1)\beta }\log\left(\frac{\omega }{1-\omega }\right)-\delta .$$

Therefore, it is guaranteed that, if $\theta \geq \theta_{0}(\omega )$, at least an ω fraction of cooperation can be expected. This condition implies that the lower bound of θ monotonically depends on β. Namely, when ω≥0.5 it increases with β, while when ω<0.5 it decreases with β.

#### (iii) Optimization problems

Bringing all these factors together, we obtain the following cost-optimization problems of institutional incentives in stochastic finite populations:

2.6 $$\min_{\theta \geq \theta_{0}(\omega )}E(\theta ),$$

where E is either $E_{r}$ or $E_{p}$, defined in (2.2), which respectively corresponds to institutional reward or punishment. We show in the electronic supplementary material that θ↦E(θ) is a smooth function on R.

### (c) Methods: evolutionary dynamics in finite populations

We adopt in our analysis the evolutionary game theory (EGT) methods for finite populations [53–55]. Herein, individuals’ pay-offs represent their *fitness* or social *success*, and evolutionary dynamics is shaped by social learning [4,43], whereby the most successful players will tend to be imitated more often by the other players. Here, social learning is modelled using the pairwise comparison rule [44], that is, a player A with fitness $f_{A}$ adopts the strategy of another player B with fitness $f_{B}$ with probability given by the Fermi function,

$$P_{A,B}={\left(1+{\text{e}}^{-\beta (f_{B}-f_{A})}\right)}^{-1},$$

where β conveniently describes the selection intensity (β=0 represents neutral drift while β→∞ represents increasingly deterministic selection).

In the absence of mutations or exploration, the end states of evolution are inevitably monomorphic: once such a state is reached, it cannot be escaped through social learning. We assume that, with a certain mutation probability, an individual switches randomly to a different strategy without imitating another individual. In addition, we assume here the small mutation limit [53,55,58]. Thus, at most two strategies are present in the population at a time. The evolutionary dynamics can be described by a Markov chain, where each state represents a homogeneous population and the transition probabilities between any two states are given by the fixation probability of a single mutant [53,55,58]. The resulting Markov chain has a stationary distribution, which describes the average time the population spends in an end state. The small mutation limit allows us to obtain an analytical form of the frequency of cooperation (see below). It is noteworthy that, although we focus here on the small mutation limit, this approach has been shown to be widely applicable to scenarios which go well beyond the strict limit of very small mutation rates [45,46,48,59].

The fixation probability of a single mutant *A* taking over a whole population with (N−1) *B* players is as follows (see [44,55,60] for details)

$$\rho_{B,A}={\left(1+\sum_{i=1}^{N-1}\prod_{j=1}^{i}\frac{T^{-}(j)}{T^{+}(j)}\right)}^{-1},$$

where $T^{\pm }(k)=(\left(N-k\right)/N)(k/N){\left[1+{\text{e}}^{\mp \beta [\Pi_{A}(k)-\Pi_{B}(k)]}\right]}^{-1}$ describes the probability of changing the number of *A* players by ± one in a time step. Specifically, when β=0, $\rho_{B,A}=1/N$, representing the transition probability at the neutral limit.

Considering the set of two strategies *C* and *D* (see [53,58] for the calculation for any number of strategies). Their stationary distribution is given by the normalized eigenvector associated with the eigenvalue 1 of the transpose of a matrix [53,58]

$$M=\left(\begin{array}{cc} 1-\rho_{C,D} & \rho_{C,D}\\ \rho_{D,C} & 1-\rho_{D,C} \end{array}\right),$$

which is $\left\{\rho_{D,C}/\left(\rho_{D,C}+\rho_{C,D}\right),\rho_{C,D}/\left(\rho_{D,C}+\rho_{C,D}\right)\right\}$. The first term is the frequency of cooperation and the second one is that of defection.

## 3. Main results

The present paper provides a rigorous analysis of the expected total cost of providing an institutional incentive (2.2) and the associated optimization problem (2.6). In this section, we state our main analytical results, theorems 3.1–3.4, and provide numerical simulations to illustrate the analytical results. The proofs of these results, which require a delicate analysis of the cost functions, are presented in the electronic supplementary material.

In the following theorems, E denotes the cost function for either institutional reward, $E_{r}$, or institutional punishment, $E_{p}$, as obtained in (2.2). Also, $H_{N}$ denotes the well-known harmonic number

3.1 $$H_{N}:=\sum_{j=1}^{N-1}\frac{1}{j}.$$

Our first main result provides qualitative properties and asymptotic limits of E.

**Theorem 3.1. (qualitative properties and asymptotic limits of total cost functions)**

(I) (*finite population estimates*) *The expected total cost of providing an incentive satisfies the following estimates for all finite populations of size* N:
  3.2 $$\frac{N^{2}\theta }{2}\left(H_{N}+\frac{1}{N-1}\right)\leq E(\theta )\leq N(N-1)\theta (H_{N}+1).$$
(II) (*infinite population limit*) *The expected total cost of providing an incentive satisfies the following asymptotic behaviour when the population size* N *tends to* +∞:
  3.3 $$\lim_{N\to +\infty }\frac{E(\theta )}{\frac{N^{2}\theta }{2}(\ln N+\gamma )}=\left\{ \begin{array}{ll} 1+{\text{e}}^{-\beta |\theta -c|} & \text{for DG},\\ 1+{\text{e}}^{-\beta |\theta -c|}{\text{e}}^{\beta c\frac{r}{n}} & \text{for PGG}, \end{array}\right.$$
  *where* γ=0.5772⋯ *is the Euler–Mascheroni constant*.
(III) (*weak selection limit*) *The expected total cost of providing an incentive satisfies the following asymptotic limit when the selection strength* β *tends to 0*:
  3.4 $$\lim_{\beta \to 0}E(\theta )=N^{2}\theta H_{N}.$$
(IV) (*strong selection limit*) *The expected total cost of providing an incentive satisfies the following asymptotic limit when the selection strength* β *tends to* +∞:
  3.5 $$\lim_{\beta \to +\infty }E_{r}(\theta )=\left\{ \begin{array}{ll} \frac{N^{2}}{2}\theta \left(\frac{1}{N-1}+H_{N}\right) & \text{for}\;\theta <-\delta ,\\ N^{2}\theta H_{N} & \text{for}\;\theta =-\delta ,\\ \frac{N^{2}}{2}\theta (1+H_{N}) & \text{for}\;\theta >-\delta \end{array}\right.$$
  *and*
  3.6 $$\lim_{\beta \to +\infty }E_{p}(\theta )=\left\{ \begin{array}{ll} \frac{N^{2}\theta }{2}(1+H_{N}) & \text{for}\;\theta <-\delta ,\\ N^{2}\theta H_{N} & \text{for}\;\theta =-\delta ,\\ \frac{N^{2}\theta }{2}\left(H_{N}+\frac{1}{N-1}\right) & \text{for}\;\theta >-\delta . \end{array}\right.$$

**Figure 1.**
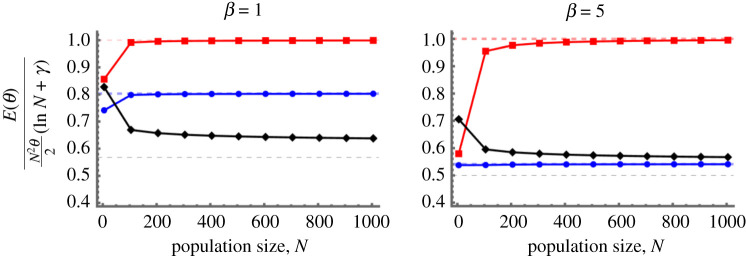
Large population size limit. We calculate numerically the expected total cost of incentive E for reward and punishment, varying population size N, for different values of θ and β. The dashed lines represent the corresponding theoretical limiting values obtained in theorem 3.1 for the large population size limit, N→+∞. We observe that numerical results are in close accordance with those obtained theoretically. Results are obtained for DG with b=2, c=1. (Online version in colour.)

The lower and upper bounds obtained in part (I) of the theorem suggest that the total expected cost function E for both reward and punishment behaves asymptotically in the order of $(N^{2}H_{N})\times \theta$ for sufficiently large N. This is confirmed in part (II), noting that $H_{N}∼\ln N$. We also show that the leading asymptotic coefficient of E depends on the game (i.e. DG or PGG) and its parameters. Hence, it is important to adopt a precise optimal value of θ (e.g. obtained by solving the optimization problem (2.6)), as a small increase in this individual incentive cost can lead to a significant increase in E, especially when the population size is large. Figure 1 numerically demonstrates this asymptotic limit.

**Figure 2.**
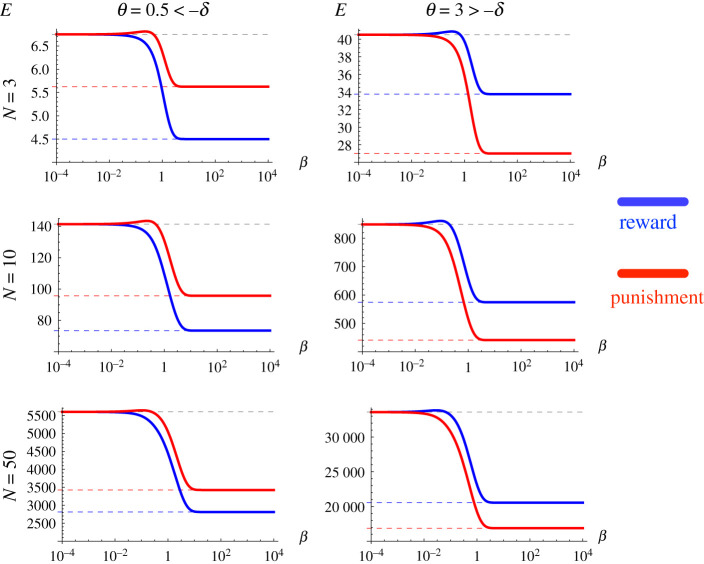
Weak and strong selection limits. We calculate numerically the total expected cost of incentive E for reward and punishment, by varying the intensity of selection, for different values of N and β. The dashed lines represent the corresponding theoretical limiting values obtained in theorem 3.1 for weak and strong selection limits. We observe that numerical results are in close accordance with those obtained theoretically. Results are obtained for DG with b=2, c=1. (Online version in colour.)

Parts (III) and (IV) of the theorem provide theoretical estimations of E under the weak (β→0) and strong (β→+∞) selection limits. For the weak selection limit, the expected total costs are the same for reward and punishment, i.e. $E_{r}(\theta )=E_{p}(\theta )$. For the strong selection limit, $E_{r}$ is smaller than, equal to or greater than $E_{p}$, depending on whether θ is smaller than, equal to or greater than −δ. Figure 2 provides numerical validation of the theoretical weak and strong selection asymptotic behaviours of E, for different population sizes N. We can observe that, for a given individual incentive cost θ, the range of E increases significantly for larger N.

Our second main result concerns the optimization problem (2.6). We show that the cost function E exhibits a phase transition when the selection intensity β varies.

**Theorem 3.2. (optimization problems and phase transition phenomenon)**

(I) (*phase transition phenomena and behaviour under the threshold*) *Define*
  $$F^{*}=\left\{ \begin{array}{ll} \min\{F(u):P(u)>0\} & \text{in the reward case},\\ \min\{\hat{F}(u):\hat{P}(u)>0\} & \text{in the punishment case}, \end{array}\right.$$
  *where* P(u) *and* F(u) *as well as* $\hat{P}$ *and* $\hat{F}$ *are defined in the electronic supplementary material* (*see* §§1 *and* 2 *there, respectively*). *There exists a threshold value* $\beta^{*}$ *given by*
  $$\beta^{*}=-\frac{F^{*}}{\delta }>0,$$
  *such that* θ↦E(θ) *is non-decreasing for all* $\beta \leq \beta^{*}$ *and is non-monotonic when* $\beta >\beta^{*}$. *As a consequence, for* $\beta \leq \beta^{*}$
  3.7 $$\min_{\theta \geq \theta_{0}}E(\theta )=E(\theta_{0}).$$
(II) (*behaviour above the threshold value*) *For* $\beta >\beta^{*}$, *the number of changes of the sign of* $E^{\prime }(\theta )$ *is at least two for all* N *and there exists an* $N_{0}$ *such that the number of changes is exactly two for* $N\leq N_{0}$. *As a consequence, for* $N\leq N_{0}$, *there exist* $\theta_{1}<\theta_{2}$ *such that, for* $\beta >\beta^{*}$, E(θ) *is increasing when* $\theta <\theta_{1}$, *decreasing when* $\theta_{1}<\theta <\theta_{2}$ *and increasing when* $\theta >\theta_{2}$. *Thus, for* $N\leq N_{0}$,
  $$\min_{\theta \geq \theta_{0}}E(\theta )=\min\{E(\theta_{0}),E(\theta_{2})\}.$$

The proofs of theorems 3.1 and 3.2 for the cases of reward and punishment are given in §§1 and 2 in the electronic supplementary material, respectively. We also provide explicit computations for N=3 and N=4 to illustrate these theorems in §3 in the electronic supplementary material. Based on numerical simulations, we conjecture that the requirement $N\leq N_{0}$ could be removed and theorem 3.2 is true for all finite N. In electronic supplementary material, figure S2, using numerical calculation we have shown that $N_{0}=100$ satisfies the conjecture, ensuring the validity of the numerical examples below. Theorem 3.2 gives rise to the following algorithm to determine the optimal value $\theta^{*}$ for $N\leq N_{0}$.

**Algorithm 3.3. (finding optimal cost of incentive $\theta^{⋆}$)**

***Inputs***: (*i*) $N\leq N_{0}$: *population size*, (*ii*) β: *intensity of selection*, (*iii*) *game and parameters: PD* (c *and* b) *or PGG* (c, r *and* n), *and* (*iv*) ω: *minimum desired cooperation level*.

(*1*) *Compute* δ {*in PD*: δ=−(c+(b/(N−1))); *in PGG*: δ=−c(1−((r(N−n))/(n(N−1))))}.
(*2*) *Compute* $\theta_{0}=(1/(N-1)\beta )\log(\omega /\left(1-\omega \right))-\delta$.
(*3*) *Compute*
  $$F^{*}=\left\{ \begin{array}{ll} \min\{F(u):P(u)>0\} & \text{in the reward case},\\ \min\{\hat{F}(u):\hat{P}(u)>0\} & \text{in the punishment case}, \end{array}\right.$$
  *where* P(u) *and* F(u), *as well as* $\hat{P}$ *and* $\hat{F}$, *are defined in the electronic supplementary material*.
(*4*) *Compute* $\beta^{*}=-(F^{*}/\delta )$.
(*5*) *If* $\beta \leq \beta^{*}$:
  $$\theta^{*}=\theta_{0},\;\min E(\theta )=E(\theta_{0}).$$
(*6*) *Otherwise* (*i.e. if* $\beta >\beta^{*}$)
  (*a*) *Compute* $u_{2}$ *that is the largest root of the equation* F(u)+βδ=0 *for the reward case or that of* $\hat{F}(u)+\beta \delta =0$ *for the punishment case*.
  (*b*) *Compute* $\theta_{2}=(\left(\log u_{2}\right)/\beta )-\delta$:
    — *if* $\theta_{2}\leq \theta_{0}$: $\theta^{*}=\theta_{0},\;\min E(\theta )=E(\theta_{0})$.
    — *Otherwise* (*if* $\theta_{2}>\theta_{0}$):
      — *if* $E(\theta_{0})\leq E(\theta_{2})$: $\theta^{*}=\theta_{0},\;\min E(\theta )=E(\theta_{0})$;
      — *if* $E(\theta_{2})<E(\theta_{0})$: $\theta^{*}=\theta_{2},\;\min E(\theta )=E(\theta_{2})$.

***Output***: $\theta^{*}$ *and* $E(\theta^{*})$.

**Figure 3.**
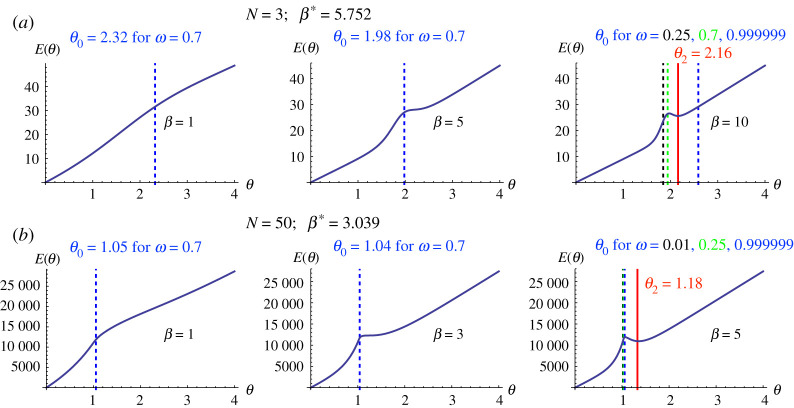
We use algorithm 3.3 to find optimal θ that minimizes E(θ) (for institutional reward) while ensuring a minimum level of cooperation ω. We also use as examples a small population size (N=3(*a*)) and a larger one (N=50(*b*)), for DG (b=1.8, c=1). (Online version in colour.)

To illustrate theorem 3.2 and algorithm 3.3, we focus on the case of reward. Figure 3 shows the cost function $E_{r}$ as a function of θ, for different values of N, β and ω to illustrate the phase transition when varying β, in a DG. We can see that, in all cases, these numerical observations are in close accordance with theoretical results. For example, with N=3 (figure 3*a*), we found $\beta^{⋆}=f^{⋆}/\delta =10.9291/1.9=5.752$. For $\beta <\beta^{⋆}$, E(θ) are increasing functions of θ. Thus, the optimal cost of incentive $\theta^{⋆}=\theta_{0}$, for a given required minimum level of cooperation ω. For example, with N=3, for β=1 to ensure at least 70% of cooperation (ω=0.7), then $\theta^{⋆}=\theta_{0}=2.32$. When $\beta \geq \beta^{⋆}$ one needs to compare $E(\theta_{0})$ and $E(\theta_{2})$. For example, with N=3, β=10: for ω=0.25 (black dashed line), then $E(\theta_{0})=23.602<25.6124=EC(\theta_{2})$, so $\theta^{⋆}=\theta_{0}=1.845$; for ω=0.7 (green dashed line), then $E(\theta_{0})=26.446>25.6124=EC(\theta_{2})$, so $\theta^{⋆}=\theta_{2}=2.16$ (red solid line); for ω=0.999999 (blue dashed line), since $\theta_{2}<\theta_{0}$, $\theta^{⋆}=\theta_{0}=2.59078$.

**Figure 4.**
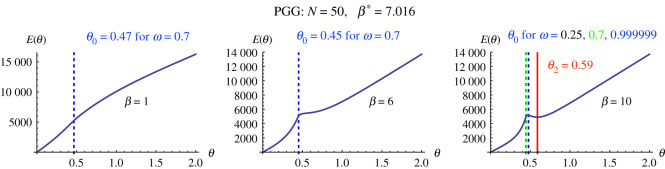
We use algorithm 3.3 to find optimal θ that minimizes E(θ) while ensuring a minimum level of cooperation ω, for PGG (r=3, n=5, c=1) with N=50. Similar observations to those for DG are obtained. (Online version in colour.)

Similarly, with a larger population size (N=50; see figure S1 in the electronic supplementary material, bottom row), we obtained $\beta^{⋆}=3.15/1.03673=3.039$. In general, similar observations are obtained as in the case of a small population size N=3. Except that, when N is large, the values of $\theta_{0}$ for different non-extreme values of minimum required cooperation ω (say, ω∈(0.01,0.99)) are very small (given the log scale of ω/(1−ω) in the formula of $\omega_{0}$). This value is also smaller than $\theta_{0}$, with a cost $E(\theta_{0})>E(\theta_{2})$, making $\theta_{2}$ the optimal cost of incentive. Similar results are obtained for PGG (figure 4). When ω is extremely high (i.e. greater than $1-10^{-k}$, for a large k) (we do not look at extremely low values since we would like to ensure at least a sufficient level of cooperation), then we can also see other scenarios where the optimal cost is $\theta_{0}$ (see figure S1 in the electronic supplementary material, bottom row). We thus can observe that for ω∈(0.01,0.99), for sufficiently large population size N and large enough β ($\beta >\beta^{⋆}+\text{a bit more}$), then the optimal value of ω is always $\theta_{2}$. Otherwise, $\theta_{0}$ is the optimal cost.

Our last result provides a comparison of the expected total costs for providing institutional reward and punishment, for different individual incentive costs θ.

**Theorem 3.4. (reward versus punishment costs)**

*The difference between the expected total costs of reward and punishment is given by*

3.8 $$(E_{r}-E_{p})(\theta )=\left\{ \begin{array}{ll} <0, & \text{for}\;\theta <-\delta ,\\ =0, & \text{for}\;\theta =-\delta ,\\ >0, & \text{for}\;\theta >-\delta . \end{array}\right.$$

*As a consequence, when* $\beta \leq \min\{\beta_{r}^{*},\beta_{p}^{*}\}$ *we have*

$$E_{r}^{*}=E_{r}(\theta_{0})\;\text{and}\;E_{p}^{*}=E_{p}(\theta_{0}).$$

*In this case,*

3.9 $$(E_{r}^{*}-E_{p}^{*})=E_{r}(\theta_{0})-E_{p}(\theta_{0})=\left\{ \begin{array}{ll} <0 & \text{for}\;\omega <0.5,\\ =0 & \text{for}\;\omega =0.5,\\ >0 & \text{for}\;\omega >0.5. \end{array}\right.$$

**Figure 5.**
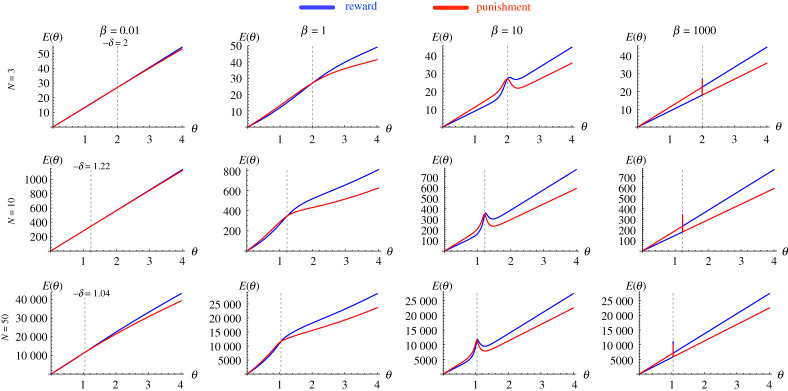
Comparison of the total costs E for reward and punishment as a function of θ, for different values of N and β. Reward is less costly than punishment ($E_{r}<E_{p}$) for small θ, and vice versa. The threshold of θ for this change was obtained analytically (see theorem 3.1), which is exactly equal to −δ. Results are obtained for DG with b=2, c=1. (Online version in colour.)

**Figure 6.**
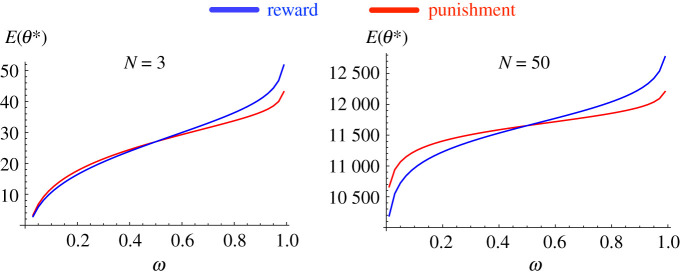
Compare the total costs E for reward and punishment at the optimal value $\theta^{⋆}$ (obtained using algorithm 3.3), by varying the minimum required level of cooperation, ω. Reward is more cost efficient for small ω, while punishment is more cost efficient when ω is larger. In both cases, the threshold is around ω=0.5. Other parameters: β=1, DG with b=2, c=1. (Online version in colour.)

The proof of theorem 3.4 is given in §3 in the electronic supplementary material. Numerical calculation in figure 5 shows the expected total costs for reward and punishment (DG), for varying θ. We observe that reward is less costly than punishment ($E_{r}<E_{p}$) for θ<−δ and vice versa when θ>−δ. It is exactly as shown analytically in theorem 3.4. This analytical result is confirmed here for different population size N and intensity of selection β. Figure 6 also confirms the second part of the theorem, where for small β, if one can choose the type of incentive to use, either reward or punishment, then the former can provide a lower cost when requiring less than 50% cooperation at minimum and the latter otherwise. This is in line with previous work showing that reward mechanisms work very well to promote cooperation in environments in which it is rare, while punishment mechanisms are better at maintaining high levels of cooperation (e.g. [28,35,52]).

## 4. Discussion

Institutional incentives such as punishment and reward provide an effective tool for promoting the evolution of cooperation in social dilemmas. Both theoretical and experimental analysis has been carried out [29,36,37,52,61–63]. However, past research usually ignores the question of how institutions’ overall spending, i.e. the total cost of providing these incentives, can be minimized, while at the same time guaranteeing a minimum desired level of cooperation over time. Answering this question allows one to estimate exactly how incentives should be provided, that is, how much to reward a cooperator and how severely to punish a wrongdoer. Existing works that consider this question usually omit the stochastic effects that drive population dynamics, namely when the intensity of selection varies.

Resorting to a stochastic evolutionary game approach for finite, well-mixed populations, we have provided theoretical results for the optimal cost of incentives that ensure a desired level of cooperation while minimizing the total budget, for a given intensity of selection, β. We show that this cost strongly depends on the value of β, owing to the existence of a phase transition in the cost functions when β varies. This behaviour is missing in works that consider a deterministic evolutionary approach [35]. The intensity of selection plays an important role in evolutionary processes. Its value differs depending on the pay-off structure (i.e. scaling game pay-off matrix by a factor is equivalent to dividing β by that factor) and is usually found to be specific for a given population, which can be estimated through behavioural experiments [45–48]. Thus, our analysis provides a way to calculate the optimal incentive cost for a given population and game pay-off matrix at hand.

With regard to theoretical importance, we characterized asymptotic behaviours of the total cost functions for both reward and punishment (namely, in the limits of a large population, weak selection and strong selection) and compared these functions for the two types of incentive. We showed that punishment is always more costly for a small (individual) incentive cost (θ) but less so when this cost is above a certain threshold. We provided an exact formula for this threshold. This result provides insights into the choice of which type of incentives to use.

In the context of institutional incentives modelling, a crucial issue is the question of how to maintain the budget for providing incentives [59,64]. The problem of who pays or contributes to the budget is a social dilemma in itself, and how to escape this dilemma is a critical research question. In this work, we focus on the question of how to optimize the budget used for the provided incentives.

There are several simplifications made for the theoretical analysis to be possible. First, in order to derive the analytical formula for the frequency of cooperation, we assumed the small mutation limit. Despite the simplified assumption, this small mutation limit approach has been shown to be widely applicable to scenarios which go well beyond the strict limit of very small mutation rates [46,48,59]. Relaxing this assumption would make the derivation of a close form for the frequency of cooperation intractable.

Second, we focused in this paper on two important cooperation dilemmas, the DG and the PGG. They have in common a useful property that the difference in (average) pay-off between a cooperator and a defector, $\delta =\Pi_{C}(i)-\Pi_{D}(i)$, does not depend on i, the number of cooperators in the population. This property allows us to simplify the fundamental matrix to a tridiagonal form and apply the techniques of matrix analysis to obtain a close form of its inverse matrix (see electronic supplementary material). In games with more complex pay-off matrices such as the PD in its general form and the collective risk game [65], the difference δ depends on i and the technique in this paper cannot be directly applied. We might consider other approaches to approximate the inverse matrix, exploiting its block structure.

## Data accessibility

This article has no additional data.
